# The Endocrine Disruptor Bisphenol A (BPA) Affects the Enteric Neurons Immunoreactive to Neuregulin 1 (NRG1) in the Enteric Nervous System of the Porcine Large Intestine

**DOI:** 10.3390/ijms21228743

**Published:** 2020-11-19

**Authors:** Kamila Szymańska, Krystyna Makowska, Jarosław Całka, Sławomir Gonkowski

**Affiliations:** 1 Department of Human Physiology and Pathophysiology, School of Medicine, Collegium Medicum, University of Warmia and Mazury in Olsztyn, Warszawska Str. 30, 10-082 Olsztyn, Poland; 2 Department of Clinical Diagnostics, Faculty of Veterinary Medicine, University of Warmia and Mazury, Oczapowskiego Str. 13, 10-719 Olsztyn, Poland; krystyna.makowska@uwm.edu.pl; 3 Department of Clinical Physiology, Faculty of Veterinary Medicine, University of Warmia and Mazury, Oczapowskiego Str. 13, 10-719 Olsztyn, Poland; jaroslaw.calka@uwm.edu.pl (J.C.); slawomir.gonkowski@uwm.edu.pl (S.G.)

**Keywords:** enteric nervous system, bisphenol A, large intestine, neuregulin 1

## Abstract

The enteric nervous system (ENS), located in the wall of the gastrointestinal (GI) tract, is characterized by complex organization and a high degree of neurochemical diversity of neurons. One of the less known active neuronal substances found in the enteric neurons is neuregulin 1 (NRG1), a factor known to be involved in the assurance of normal development of the nervous system. During the study, made up using the double immunofluorescence technique, the presence of NRG1 in the ENS of the selected segment of porcine large intestine (caecum, ascending and descending colon) was observed in physiological conditions, as well as under the impact of low and high doses of bisphenol A (BPA) which is commonly used in the production of plastics. In control animals in all types of the enteric plexuses, the percentage of NRG1-positive neurons oscillated around 20% of all neurons. The administration of BPA caused an increase in the number of NRG1-positive neurons in all types of the enteric plexuses and in all segments of the large intestine studied. The most visible changes were noted in the inner submucous plexus of the ascending colon, where in animals treated with high doses of BPA, the percentage of NRG1-positive neurons amounted to above 45% of all neuronal cells. The mechanisms of observed changes are not entirely clear, but probably result from neurotoxic, neurodegenerative and/or proinflammatory activity of BPA and are protective and adaptive in nature.

## 1. Introduction

Neuregulin 1 (NRG1) was described for the first time in 1992 as a 44-kD glycoprotein purified from the medium of a human breast tumor cell line [1]. This substance, together with three other neuregulins (NRG2, NRG3 and NRG4), belongs to a group of structurally related substances which form part of the epithelial growth factor family proteins and may affect the ErbB receptors [2], whereby NRG1 shows the highest affinity to ErbB 2 and ErbB 3 receptors [3]. ErbB receptors are important to the development of the living organism and their stimulation generally results in apoptosis inhibition, intensification of angiogenesis and enhancement of cell survivability [4].

Till now, the presence of NRG1 has been reported in various internal organs and systems, including the nervous system of various animal species (for a review, see [5]). As regards the nervous system, the majority of previous studies concern distribution and functions of NRG1 in the central nervous system. This substance has been found in the brain of various animal species, including, among others, human [6], rat [7], mouse [8] and goose [9]. Based on previous studies, it is known that NRG1 in the central nervous system is involved in hypothalamic control of sexual maturation in rats [10], participates in mechanisms connected with oligodendrocytes survivability and differentiation in mice [11] and also regulates the higher nervous functions in rats (including memory and learning) [12].

Contrary to the central nervous system, the knowledge about the distribution and functions of NRG1 in the peripheral nervous system is more scarce and fragmentary. It is known that NRG1 is present in the dorsal root ganglia in rats and mice [13,14], in nervous structures supplying the digestive tract in humans [3] and in nerves located in the liver and uterus of the domestic pig [15,16].

It should be underlined that NRG1 in the gastrointestinal (GI) tract is present not only in the neuronal structures, but also in other parts of the intestinal wall, including mucosal and muscular layers. Previous studies concerning distribution of NRG1 in the GI tract of various animal species are summarized in Table 1. Moreover, a gene responsible for NRG1 synthesis has been found in the human GI tract [17,18,19,20].

Investigations conducted on rodents have described that NRG1 in the peripheral nervous system participates in Schwann cells differentiation, axonal preservation and may show neuroprotective activity affecting survivability and regeneration of Schwann cells in rats [29,30]. Moreover, it is known that NRG1 enhances muscle re-innervation [31], affects the neurotransmitter receptors and ensures the proper functioning of peripheral synapses [32]. Regarding the innervation of the GI tract, it is known that NRG1 plays a key role in the development of the enteric nervous system (ENS), which has been confirmed by studies conducted on mice [33,34] and zebrafish [28]. In turn, observations conducted on humans have indicated that NRG1 is involved in pathological processes connected with intestinal diseases resulting from incorrect organization of the enteric ganglia, including Hirschsprung’s disease and diverticular disease [18,22,35].

**Figure 1:**
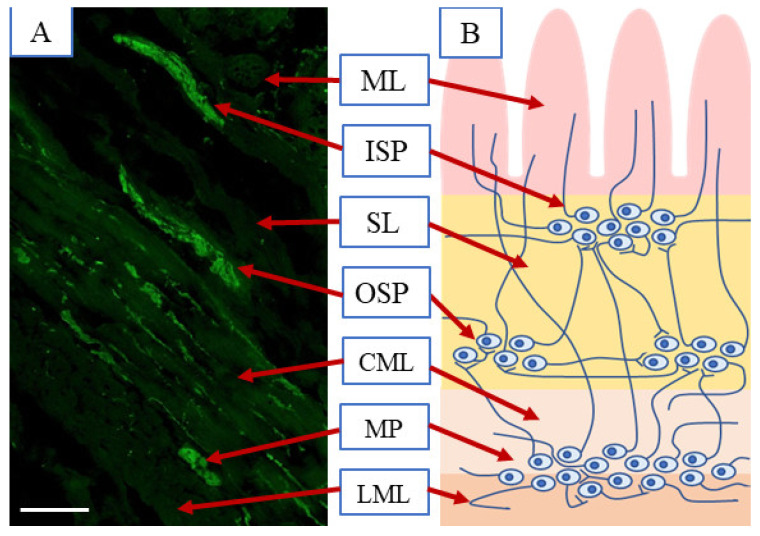
Microscopic view of the porcine large intestine—caecum (scale bar 100 µm) after labelling with panneuronal marker protein gene product 9.5—PGP 9.5 (**A**) and scheme (**B**) of the enteric nervous system: ML—mucosal layer, ISP—inner submucous plexus, SL—submucosal layer, OSP—outer submucous plexus, CML—circular muscular layer, LML—longitudinal muscular layer.

It should be underlined that the ENS is the most unique part of the peripheral nervous system. It is located in the wall of the GI tract from the esophagus to the rectum and is characterized by a high number of neuronal cells, complex structure and significant independence from the central nervous system, thanks to which it is often called “the second” or “the intestinal” brain [36]. The construction of the ENS depends on the animal species and segment of the gastrointestinal tract. In the large intestine of domestic pig, the ENS (Figure 1) is built of intramural neuronal ganglia, which are interconnected with a dense network of nerves, and is formed in three types of enteric plexuses. These are: the myenteric plexus (MP) located in the muscular layer between longitudinal and circular muscular coats, the outer submucous plexus (OSP) placed on the internal side of the circular muscles layer and the inner submucous plexus (ISP) located near the lamina muscularis mucosae [37]. 

The enteric neurons are characterized by the high degree of diversity with regard to neurochemical characterization. Apart from acetylcholine—the main neuromediator in the ENS, enteric neurons show the presence of several dozen other active substances, of which the vast majority are neuromediators and/or neuromodulators [37,38,39]. Moreover, it is known that neurochemical characterization of the enteric neurons may be subjected to changes under the impact of physiological and pathological stimuli including, among others, growth and puberty, diet changes, gastrointestinal or systemic diseases, and these changes are primarily of an adaptive and protective nature [37,39,40].

As mentioned above, one of the substances previously described in the gastrointestinal nervous structures is NRG1 [3]. However, the knowledge about NRG1 distribution in particular types of the enteric plexuses and its functions in the intestinal innervation is rather scarce [3], and there are no studies concerning changes in the number of NRG1-positive enteric neurons under toxic factors.

For this reason, the aim of the present experiment was to study the number of NRG1-positive neurons in the selected parts of the porcine large intestine in physiological conditions and after the impact of various doses of bisphenol A (BPA). BPA is an organic synthetic substance which is commonly used in the production of plastics all over the world [41]. It is present in a wide range of everyday objects, such as bottles, food containers, household goods, toys, furnishing, dental materials and many others [41,42]. BPA may be released from plastics, penetrate into water and food and harmfully affect living organisms.

BPA as an endocrine disruptor shows adverse effect on various internal organs and systems. In the reproductive system BPA causes changes in the uterus manifested by the increase in the thickness of the endometrium and number of fibroblasts, as well as by the inhibition of apoptosis [43,44]. These changes are accompanied by disturbances in estrus cycle, embryo implantation after fertilization, motor activity of the uterus, sex hormones levels and secretion of active substances by nervous structures supplying the uterine [45,46,47,48]. Exposure to BPA may contribute to development of endometriosis and cervical cancer [49,50].

In turn, exposure to BPA in the nervous system results in the disorders of synaptogenesis, disturbances in the growth and development of neurites and axons, changes in ion transport through neuronal cell membrane, modification of neurochemical properties of neuronal cells and exacerbation of neurodegenerative processes [51,52,53]. It is also known that BPA affects cognitive functions of the brain, impairing processes connected with behavior, memory and ability to learn [54,55]. Some studies suggest correlations between the exposure to BPA and autism and/or neurodegenerative diseases [56].

Since BPA enters the body mainly with food, the GI tract is particularly vulnerable to the impact of this substance. Previous studies have shown that BPA causes damage of the intestinal barrier functions and increases intestinal permeability, as well as influences on the sensory stimuli conduction in the GI tract [57,58]. Moreover, BPA enhances reactions connected with oxidative stress, apoptosis, dysfunctions of mitochondria and inflammatory processes in the mucosal layer in the intestine [59]. BPA also modifies the neurochemical profile of the enteric neurons and composition of intestinal flora, what can contribute to various gastrointestinal diseases [60,61].

Of course, BPA affects also other internal organs, including, among others, the liver [62], heart [63], endocrine glands [64], kidneys [65] and lungs [66], leading to disturbances in their functioning [43,44]. In turn, epidemiological studies on human population have shown correlation between the degree of exposure to BPA and risk of obesity, diabetes, hypertension, cancer and many other pathological processes [67,68].

However, many aspects of the impact of BPA on the living organism still remain not completely explained. One of them is the influence of BPA on the ENS in the large intestine. It should be pointed out that due to the transformation of BPA in the GI tract it is very exposed to the impact of this substance. Namely, it is known that the majority of BPA which gets into the GI tract is absorbed in the proximal part of the jejunum and subjected to glucuronidation in the enterocytes [69]. Glucuronidation is considered to be a form of body’s defense against adverse effects of BPA, because metabolite formed in this process—BPA-glucuronide (BPA-GA) has smaller estrogenic activity [69]. The vast majority of BPA-GA is transported again to the lumen of the intestine. In the large intestine (mainly under the influence of microbiota in the caecum) BPA-GA is subjected to renewed deconjugation to free BPA [69]. Therefore, the wall of the large intestine is strongly exposed to adverse effects of this substance.

The aim of the present study was not only to describe for the first time the NRG1-positive enteric neurons in the porcine large intestine, but also the influence of BPA on this neuronal population. The obtained results will allow to enrich the knowledge about neurochemical characterization of the enteric neurons under physiological conditions, as well as about changes within the ENS appearing under the impact of various doses of BPA. It should be underlined that the domestic pig, due to similarities in neurochemical and anatomical properties of the ENS to human intestinal innervation, seems to be a good animal model to investigate the impact of toxic substances on the human GI tract [70]. Therefore, the obtained results may also contribute to the better understanding the influence of BPA on the human GI tract.

## 2. Results

**Figure 2:**
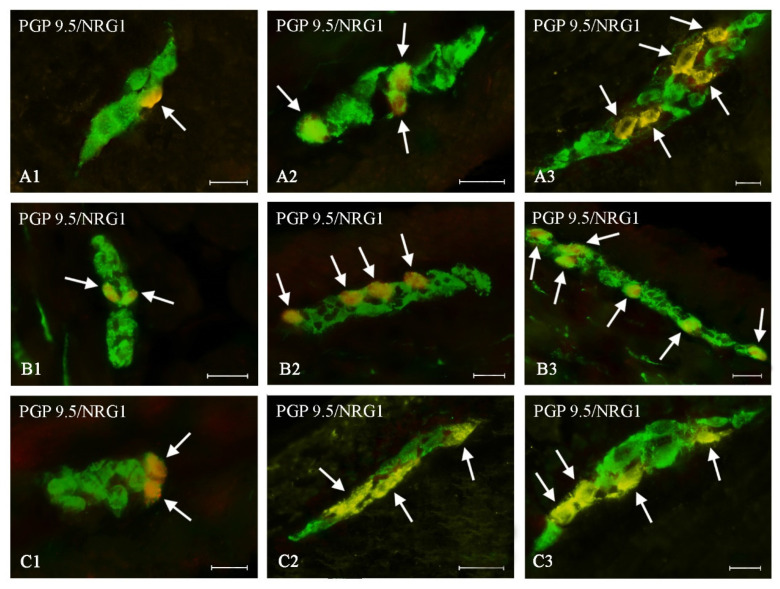
Myenteric plexus of the porcine large intestine: (**A**)—caecum, (**B**)—ascending colon, (**C**)—descending colon in various groups of animals: (1) control animals, (2) animals treated with BPA at a dose of 0.05 mg/kg b.w./day, (3) animals treated with BPA at a dose of 0.5 mg/kg b.w./day. Neurons showing co-localization of PGP 9.5 and NRG1 are indicated with arrows. The scale bar is 20 µm.

**Figure 3:**
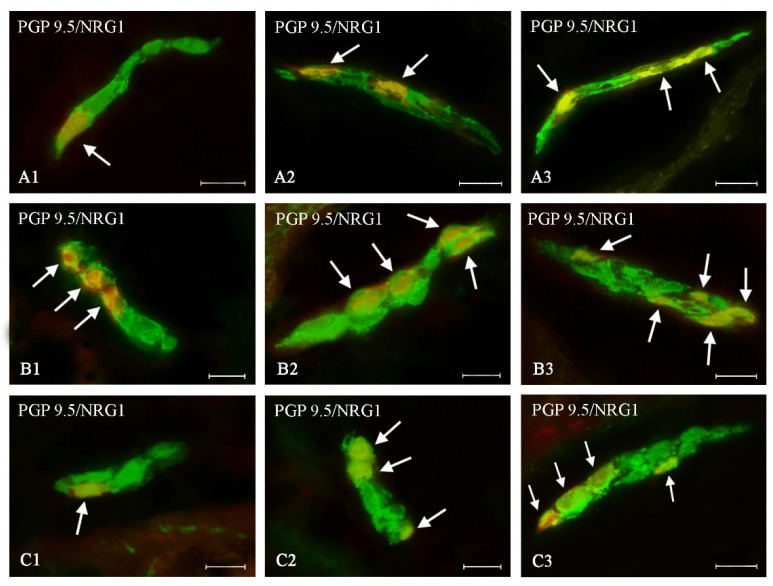
Outer submucous plexus of the porcine large intestine: (**A**)—caecum, (**B**)—ascending colon, (**C**)—descending colon in various groups of animals: (1) control animals, (2) animals treated with BPA at a dose of 0.05 mg/kg b.w./day, (3) animals treated with BPA at a dose of 0.5 mg/kg b.w./day. Neurons showing co-localization of PGP 9.5 and NRG1 are indicated with arrows. The scale bar is 20 µm.

**Figure 4:**
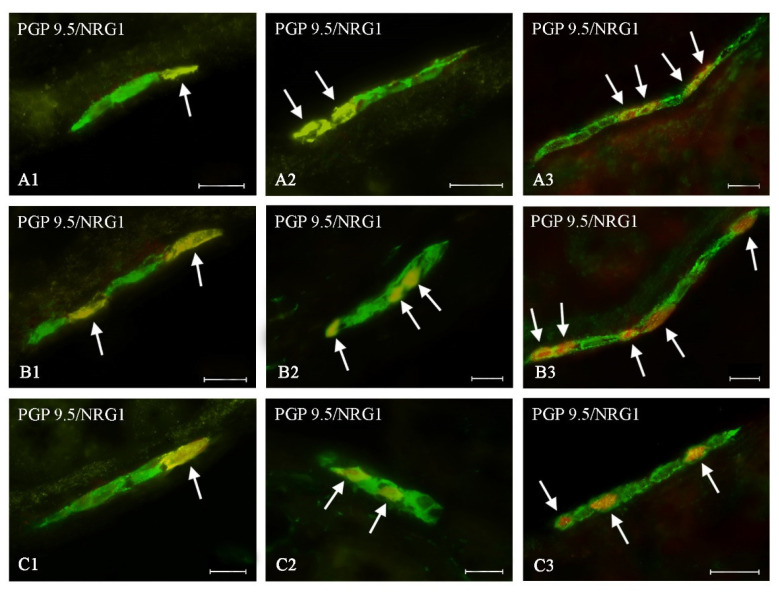
Inner submucous plexus of the porcine large intestine: (**A**)—caecum, (**B**)—ascending colon, (**C**)—descending colon in various groups of animals: (1) control animals, (2) animals treated with BPA at a dose of 0.05 mg/kg b.w./day, (3) animals treated with BPA at a dose of 0.5 mg/kg b.w./day. Neurons showing co-localization of PGP 9.5 and NRG1 are indicated with arrows. The scale bar is 20 µm.

During the present study, neuronal cells immunoreactive to NRG1 were observed in all types of enteric plexuses of all segments of the large intestine studied, both in control animals and in pigs treated with BPA (Table 2, Figure 2, Figure 3 and Figure 4). On the other hand, the presence of NRG1 was not observed in the nerve fibers located within the intestinal wall, as well as in cells immunonegative to protein gene product 9.5 (PGP 9.5- used as a panneuronal marker) located in the enteric ganglia.

### 2.1. NRG1-Positive Enteric Neurons under Physiological Condition

Under physiological conditions, the smallest number of NRG1-like immunoreactive (LI) neurons was noted in the caecum, where the percentage of NRG1-positive neurons fluctuated from 19.63 ± 0.08% of all cells immunoreactive to PGP 9.5 in the OSP to 22.43 ± 0.16% in the MP (Table 2). In the ascending colon the percentage of NRG1-positive neurons fluctuated from 22.73 ± 0.17% in the ISP to 27.49 ± 0.19% in the OSP (Table 2). In the MP and OSP within the descending colon, the number of cells immunoreactive to NRG1 was slightly lower than in the ascending colon and slightly higher than in the caecum (Table 2). In turn, in the ISP, the number of NRG1-positive cells was lower and amounted to 19.85 ± 0.28% of all cells immunoreactive to PGP 9.5 (Table 2, Figure 4).

### 2.2. The Influence of BPA on the Number of NRG1-Positive Enteric Neurons

Both doses of BPA resulted in an increase in the number of NRG1-LI neurons in all types of the enteric plexuses located in all studied segments of the large intestines (Table 2). The intensity of changes depended on the type of enteric plexus and the part of the intestine, but changes were more visible in animals treated with a higher dose of BPA.

#### 2.2.1. Caecum

In the MP (Figure 2A) BPA caused an increase in the percentage of NRG1-LI neurons to 32.64 ± 0.17% (by about 10 percentage points (pp)) and to 43.10 ± 0.15% (by about 20 pp) under lower and higher doses, respectively (Figure 2). A similar intensity of changes was noted in the caecal ISP (Table 2, Figure 4A). Less visible changes were noted in the OSP (Figure 3A), in which the percentage of NRG1-LI neurons achieved 24.77 ± 0.29% in animals treated with a lower dose of BPA (an increase of about 5 pp in comparison with the control animals) and 30.62 ± 0.23% in animals receiving higher doses of BPA (an increase of about 11 pp) (Table 2).

#### 2.2.2. Ascending Colon

In the ascending colon, the most visible changes were noted in the ISP, where under a lower dose of BPA the percentage of NRG1-LI neurons increased to 38.75 ± 0.41% (by about 16 pp), and under higher dose of BPA—to 45.55 ± 0.18% (by about 23 pp) (Figure 4B). In other types of enteric plexuses located in the ascending colon, changes were slightly less visible (Table 2). Moreover changes noted in the MP (Figure 2B) were similar to those observed in the OSP (Figure 3B).

#### 2.2.3. Descending Colon

In the descending colon, changes in the number of NRG1-LI neurons noted in the present study were generally less visible than those noted in other segments of the large intestine. This particularly concerned MP and ISP (Table 2). In the former type of the enteric plexus, a lower dose of BPA caused an increase in the number of NRG1-LI neurons to 28.99 ± 0.31% (by only about 4 pp), and a higher dose of BPA resulted in an increase in the number of such neurons to 37.11 ± 0.12% (by about 13 pp) (Figure 2C). In the ISP (Figure 4C) the changes were slightly more visible—an increase by about 6 pp and 17 pp in comparison to control animals) in pigs treated with lower and higher doses of BPA, respectively (Table 2). In turn, changes noted in the OSP of the descending colon (Figure 3C) were similar to those noted in the caecal OSP (Table 2).

### 2.3. The Size of NRG1-Positive Enteric Neurons in the Large Intestine under Physiological Conditions and after Administration of BPA

The size of enteric neurons immunoreactive to NRG 1 was generally similar in all studied fragments of the large intestine, but differed among particular types of the enteric plexuses (Table 3).

Under physiological conditions the average surface area of NRG1-positive neurons in the MP ranged from 90.68 ± 0.25 µm^2^ in the descending colon to 92.05 ± 0.35 µm^2^ in the caecum. In the OSP and ISP neurons immunoreactive to NRG 1 were smaller. In the OSP the average surface area ranged from 64.27 ± 0.27 µm^2^ in the ascending colon to 65.20 ± 0.32 µm^2^ in the caecum, and in the ISP—from 58.03 ± 0.15 µm^2^ in the descending colon to 59.25 ± 0.27 µm^2^ in the caecum (Table 3). Both lower and higher dose of BPA caused clear increase in the size of NRG1-positive neurons in all types of the enteric plexuses and all large intestine fragments studied (Table 3). Changes were more visible under the impact of the higher doses of BPA and the clearest increase of average surface area of NRG1-positive neurons was found in the MP of the descending colon, where this value increased from 90.68 ± 0.25 µm^2^ in the control animals to 104.77 ± 0.27 µm^2^ in animals treated with higher dose of BPA.

### 2.4. The Influence of BPA on the Total Number of the Enteric Neurons

The influence of BPA on the total number of the enteric neurons was noted during the present study. In animals treated with both doses of BPA total number of neurons in each type of the enteric neurons were lower in comparison to control animals. The most visible changes were observed under the higher dose of BPA in the myenteric plexus of the caecum and the ascending colon, where the average total number of enteric neurons decreased from 1086.00 ± 5.89 to 1006.60 ± 5.36 and from 1077.20 ± 5.99 to 996.20 ± 3.40, respectively (Table 4).

## 3. Discussion

The results obtained in the present study show that NRG1 is present in a relatively large population in all types of plexuses of the enteric nervous system in the porcine large intestine. These observations are in agreement with previous studies, where NRG1 was observed in the human ENS [3].

Comparing the results obtained in the domestic pig during the present study with the results of previous studies, it can be concluded that the distribution of NRG1 in the ENS clearly depends on the mammal species. Although the similarities in the organization of the ENS and neurochemical characterization of the enteric neurons between humans and the domestic pig are relatively well known [71], the differences in the localization of NRG1 in the intestinal innervation between these species are visible. In particular, the present study showed that NRG1 in pigs was located in at least 20% of all neurons in each type of the enteric plexus and was not present in nerve fibers in the intestinal wall. In turn, previous studies in humans indicated that this substance occurs not only in the myenteric and submucosal plexuses (in neuronal and glial cells), but also in nerve fibers located within the intestinal muscular layer (in nerve fibers and muscle cell nuclei) [3]. Moreover, as mentioned above, NRG1 has also been noted in glial cells located in the enteric ganglia in humans [3], whereas in the present study the occurrence of this substance in PGP 9.5-negative cells located in the enteric ganglia has not been found. Since cells in the enteric ganglia, that do not contain panneuronal marker are glial cells, the observation strongly suggests that NRG1 is not present in the enteric glial cells in juvenile domestic pigs. It should be underlined that differences in distribution of NRG1 in the GI tract between human [3] and domestic pig (this study) may result from not only interspecies differences, but also from various age of individuals included into the study. Namely, in the case of humans, tissues were obtained from persons at the age of average 75 years [3], whilst the present study has been performed on juvenile animals, and it is relatively well known that the ENS undergoes substantial changes during ageing and maturation [72]. To establish if the domestic pig is a good animal model to study functions and distribution of NRG 1 in the ENS, the further studies are necessary. However, results obtained in previous investigations significantly differ from that noted in humans [3]. This suggests that the pig cannot be used as an animal model for NRG 1 functions in the human intestine, although the organization of the ENS in human and domestic pig in many respects are similar. 

The occurrence of NRG1 in neurons located in all types of the enteric plexuses noted in the present study strongly suggests that this substance is involved in a wide range of intestinal regulatory processes connected both with motility, as well as secretory activity of the large intestine. It should be noted that the current knowledge concerning the functions of NRG1 located in the ENS is not extensive, although it is known that this substance may be involved in the development of the ENS through the enhancing of growth and differentiation of the enteric neurons during ontogenesis, what has been indicated during investigations performed on rat enteric nerve cultures [3] and zebrafish [28]. Moreover, studies performed on the chicken embryos [73] and mice [33,34] have shown that NRG1 affects the development of Schwann cells, neurite outgrowth and synaptogenesis (for review see [74]). The important roles of NRG1 in the development of the ENS are supported by the fact that in laboratory animals deprived of NRG1 or ErbB receptors, a reduction in the number of synapses and enteric ganglia has been noted [75,76]. Moreover, it is known that disturbances of NRG1 expression in the intestinal nervous structures occur during gastrointestinal diseases, including diventricular disease and Hirschsprung’s disease [18,22,35]. Previous studies also strongly suggest the roles of NRG1 localized in the neuronal cells of the myenteric plexus in regulatory processes connected with gastrointestinal motility [3].

The results obtained during the present study have shown that administration of BPA results in the changes on the number of NRG 1-positive enteric neurons in the large intestine. It should be pointed out that the large intestine is a tissue with high number of estrogen receptors. Both estrogen receptors α and β have been found in the large intestine [77,78,79]. They are located in the mucosal layer, both in cells located in the submucosal layer identified as macrophages, as well as in the neuronal cells of the ENS [78,79] and they are involved in many processes including, among others, intestinal motility, inflammatory reactions, cell proliferation, regulation of mitochondrial function and protective processes (for review, see [80]). Since BPA is known as a ligand of the estrogen receptors, and previous studies also linked the levels of NRG 1 expression with endocrine resistance [81,82], the changes noted in the present study may result from endocrine disruption caused by BPA.

However, the elucidation of the exact mechanisms of noted changes is not clear. They may be connected with the neurotoxic and neurodegenerative effects of BPA. These effects are known from the previous studies, where BPA-induced disturbances in synaptogenesis, development of neural dendrites, calcium homeostasis and production of active substances in the nervous structures have been described [53,83,84,85]. In the central nervous system, the exposure to BPA also results in dysfunctions in higher neural activity, such as memorizing and learning, which may occur even a long time after contact with this substance [86,87]. The knowledge of the influence of BPA on the peripheral nervous system is more limited than on the central nervous system. It is known that BPA inhibits the voltage-gated sodium channels in dorsal root ganglia and may change the neurochemical characterization in autonomic nervous structures supplying various internal organs [60,88,89,90,91]. The decrease in total number of neurons noted in the present study under the impact of BPA also confirms neurotoxic properties of this substance. Taking into account the neurotoxicity of BPA, on the one hand, and the positive influence of NRG1 on neuronal cells and its neuroprotective effects on the other [8], it can be assumed that changes noted in the present study are connected with neuroprotective and adaptive reactions under the impact of BPA.

It cannot be excluded that the increase in the percentage of NRG1-positive neurons in respect to total number of PGP 9.5-immunoreactive cells is caused by neurotoxic effects of BPA and BPA-induced decrease in the total number of the enteric neurons noted in the present study. Such situation would also indicate the neuroprotective properties of NRG1, which caused higher survivability of neurons containing this substance compared to other neuronal populations. This is all the more likely that recent studies confirm that NRG 1 plays a key role in the intestinal stem cells proliferation and regeneration of epithelial cell after damage [27]. The mentioned investigation has shown that after the influence of pathological factors leading to the injury of epithelial cells in the mouse intestine, a robust increase in NRG 1 expression has been noted. Moreover, the same authors have indicated that NRG1 is an essential factor to cellular proliferation and homeostasis maintenance during the regenerative processes in the intestinal mucosal layer following damage [27]. The increase in the number of NRG 1-positive enteric neurons noted in the present study may be a sign of such protective reactions.

Further reasons for the fluctuations of the percentage of NRG1-positive neurons noted in the present experiment may be connected with the pro-inflammatory activity of BPA or with the direct influence of this substance on intestinal motility. The former is supported by the fact that BPA is a relatively well-known pro-inflammatory factor affecting the intestinal mucosal layer [58,92] and immune system leading to changes in cytokines levels [93], and the anti-inflammatory activity of NRG1 has already been described [94]. It cannot be excluded that changes in the neurochemical characterization of the enteric neurons are the first signs of subclinical inflammatory process, especially that similar situation has been noted in duodenum [95].

In turn, the changes noted in the present study are supported by the fact that BPA inhibits the activity of the intestinal muscles [96] and NRG1 is an important factor involved both in the formation and functionality of the neuromuscular junction [97], as well as being a modulator of muscle metabolism [98]. Therefore, the increase in the number of NRG1-positive neurons (especially in the myenteric plexus, which is mainly responsible for the intestinal motility) might be the answer to BPA-induced inhibition of motility. In turn, the connection of the observed changes with sensory and pain stimuli conduction is not very likely despite the participation of NRG1 in this process [13], because the doses of BPA used in the present study were rather low and no symptoms associated with pain or inflammation in the experimental animals were observed. It should be pointed out that BPA in both doses used in this experiment not only changed the percentage of NRG1-positive neurons (in relation to total number of the enteric neurons), but also affected the morphology of neuronal cells immunoreactive to NRG1. This impact consisted in the increase of the average surface area of NRG1-positive neuronal cells. Previous studies have reported that BPA may affect the size of neuronal cells in the central nervous system, and the character of changes depends on the nervous structures studied and the age and the sex of experimental animals. For example, it is known that BPA causes the decrease the soma size of motoneurons in the lumbar spinal cord [99], but increases of volume of sexually dimorphic nucleus of the preoptic area in male (but not in female) rats [100]. However, the exact mechanisms responsible for the influence of BPA on neuronal size still remain not clear.

On the other hand, differences in the average size of NRG1-positive cells between control animals and pigs receiving BPA may suggest that under the impact of BPA NRG1 starts to be synthetized and ectopically expressed in population of neurons, which under physiological conditions do not show such abilities. 

Interestingly, clear changes were noted in animals treated with a lower dose of BPA (0.05 mg/kg b.w./day). Until recently, this dose was fixed as a tolerable daily intake (TDI) by the European Food Safety Authority (EFSA) [101]. Admittedly, in 2015 the EFSA temporarily reduced the TDI for BPA to 4 µg/kg b.w./day [102] making the final decision dependent on further studies, but in many countries the TDI or the reference dose for BPA was set at 0.05 mg/kg b.w./day [103]. The results obtained during the present study, together with previous studies in which the influence of BPA at the dose of 0.05 mg/kg b.w./day on the neurochemical characterization of neurons supplying the uterus, liver and GI tract was reported [60,89,90], indicate that such doses are not neutral for a living organism. Of course, it should be underlined that the dose of 0.05 mg/kg b.w./day is significantly higher than the doses on which people are exposed in everyday life. Since BPA may affect the human body in a different way (GI tract, lungs and skin), and the degree of exposure clearly depends on various factors including not only part of the world or environmental pollution but also diet and chosen profession [41,42], the establishing the real daily exposure to this substance is relatively difficult. Nevertheless, current studies describe two methods of estimation of exposure to BPA, based on urinary excretion and wastewater-based epidemiology [104]. According to the first method, the average human exposure to BPA has been assessed at the level of 2.53 µg/day (the highest exposure in some regions amounted about 14.5 µg/day), and according to the second method—513.73 µg/day [104]. Nevertheless, on the other hand, in some cases, the daily exposure of humans to BPA may be close to, or even higher, than the dose of 0.05 mg/kg b.w./day. For example, it has been found that in humans with reconstructions of molar teeth crowns, the release of BPA from the dental fillings may reach 30 mg/day [105,106]. Such situation has been confirmed by evaluation of the degree of exposure based on wastewater-based epidemiology, which indicated that in some regions human exposure to BPA amounted to even 11,554.50 µg/day [104]. To sum up, the present study has shown that dose of BPA, which until recently has been established as TDI, is not neutral for the living organism, and the decision of EFSA about reduction of TDI dose for BPA was correct.

## 4. Materials and Methods

### 4.1. Experimental Animals and Administration of BPA

The present experiment was performed on 15 immature female pigs of the Piétrain x Duroc breed which were about 8 weeks old and bought from a commercial farm. During the study, the animals were kept in the animal house of the Faculty of Veterinary Medicine, University of Warmia and Mazury in Olsztyn (Poland). After the transport, the pigs were randomly divided and placed in pens (five animals in each) suitable for animal species and ages. The animals were fed with typical commercial feed meant for piglets and had unlimited access to drinking water. All procedures conducted in the present experiment received the approval of the Local Ethics Committee responsible for experimental animals in Olsztyn (Poland) (decision numbers 28/2013 of 22 May 2013 and 65/2013/DLZ of 27 November 2013).

After a five-day adaptive period, the administration of BPA was started. BPA was given orally in capsules during the morning feeding. Five animals (Experimental I—Ex I group) received BPA at a dose of 0.05 mg/kg body weight (b.w.)/day. The next five animals (Experimental II—Ex II group) were treated with a ten-fold higher dose of BPA (0.5 mg/kg b.w./day). The lower dose of BPA administered to animals of Ex I group is a dose that until recently, it was fixed as a tolerable daily intake (TDI) in the countries of the European Union [101], and therefore, it was considered to be safe for humans and animals. Moreover, this dose is still recognized as a TDI or reference dose for BPA in some countries of the world [103].

All the other pigs (in the number of 5) constituted the control group (C group) and received empty capsules in the same way as animals in Ex I and Ex II groups. All animals received capsules for 28 days. After this period, all animals were pre-medicated with Stresnil (Janssen, Beerse, Belgium, 75 μL/kg of b.w., given intramuscularly) and after about 30 min. they were subjected to euthanasia by an overdose of sodium thiopental (Thiopental, Sandoz, Kundl, Austria).

### 4.2. Tissue Collection

Immediately after death, fragments (ca. 3 cm long each) of the selected parts of the large intestine were collected. The exact locations of the collected fragments were as follows: (1) caecum—fragment located 7 cm from the ileocecal valve, (2) ascending colon—fragment of the apex located between centripetal and centrifugal turns, (3) descending colon—fragment located where nerves from the inferior mesenteric ganglia supply the intestine. Immediately after collection, intestinal fragments were fixated in 4% buffered paraformaldehyde (pH 7.4) for 1 h at room temperature (rt) and were rinsed in phosphate buffer for three days at 5 °C (buffer was changed every day). After this time, the fragments of the intestine were put into 18% phosphate-buffered sucrose and storaged at 5 °C for at least three weeks. The collected tissues were then frozen at −22 °C and cut perpendicular to the gut lumen into 14-μm-thick sections using a cryostat (HM 525, Microm International, Dreieich, Germany). The obtained slices were mounted on microscopic slides. Slides with gut slices were stored at −22 °C until further evaluation.

### 4.3. Immunofluorescence Method

The fragments of the intestine were subjected to the double immunofluorescence method previously described by Gonkowski and Calka [107] and it consists of the following stages: (1) drying the slides with gut slices for 1 h (rt), (2) incubation with a solution containing 10% normal goat serum, 0.1% bovine serum albumin, 0.01%NaN3, 0.25% Triton x-100 and 0.05% thimerosal in PBS in a humid chamber for 1 h. (rt), (3) incubation with a mixture of the antibody directed against pan-neuronal marker protein gene product 9.5—PGP 9.5 (mouse anti-human PGP 9.5 antibody that it cross-reacts with the pig, catalogue no. 7863-2004 obtained from Bio-Rad, Hercules, CA, USA, work dilution 1:1000) and the antibody directed against NRG1 (rabbit anti-human NRG1 antibody, (AA 198–229) obtained from Antibodies-online, Aachen, Germany, work dilution 1:1000) in a humid chamber (overnight, rt), (4) incubation with a mixture of species-specific secondary antibodies conjugated with two different fluorochromes: Alexa Fluor 488 (donkey anti-mouse IgG, Invitrogen, Carlsbad, CA, USA, working dilution 1:1000) and Alexa Fluor 546 (donkey anti-rabbit IgG, Invitrogen, working dilution 1:1000) in a humid chamber (1 h, rt), (5) covering the intestinal fragments with buffered glycerol and “closing” the slices with a cover slips. The intestinal fragments were rinsed in PBS (3 × 10 min.) between the particular above-mentioned stages of labeling. In order to avoid non-specific NRG 1 labeling, typical tests of antibody were performed during the present study. They included: (1) pre-absorption test, in which 1 mL of anti NRG 1 antibody in working dilution was incubated (overnight) with 100 µg of appropriate antigen (Neuregulin 1 Antibody Blocking Peptide, LS-E8137-500, LSBio Seattle, WA, USA) and then used as a primary antibody in the immunofluorescence method, (2) omission test, in which anti-NRG 1 antibody was replaced with labelling with 1% normal horse serum in PBS. Mentioned above tests prevented NRG 1-positive labelling in the porcine large intestine.

### 4.4. Cell Counting

Labelled fragments of the whole intestinal wall were evaluated using a BX51 microscope (Olympus, Tokyo, Japan) equipped with epi-fluorescence and appropriate filter sets for Alexa Fluor 488 (B1 module, excitation filter 450–480 nm, barrier filter 515 nm, UMNB2, Olympus) and Alexa Fluor 546 (G1 module excitation filter 510–550 nm, barrier filter 590 nm, U-N31046, Olympus). CellSens (Olympus) was used as an imaging software. The determination of the percentage of enteric neurons immunoreactive to NRG1 involved the evaluation of at least 500 PGP 9.5-LI neurons within each type of the enteric plexus in each animal for the presence of NRG1, in which the number of PGP 9.5-positive cells was considered as 100%. Neuronal cells were counted in the MP—located between longitudinal and circular muscles layer, OSP—located on the internal side of the circular muscles layer and ISP located near the lamina muscularis mucosae (Figure 1). The classification of the type of enteric ganglia was carried out in the whole intestinal wall based on their position. To prevent double-counting the same cells, intestinal slices included in the study were located at least 200 µm apart from each other.

Moreover, to study if BPA affects the general number of enteric neurons, the number of all PGP 9.5-positive neuronal cells was counted in the enteric ganglia. Neurons were counted in six slices (located at least 400 µm apart from each other) of each part of the large intestine and each animal.

### 4.5. Evaluation of Average Surface of NRG1-Positive Neurons

The average surface area of neurons immunoreactive to NRG1 (performed on 100 randomly selected NRG1-positive neurons from each type of the enteric plexus and each animal) was evaluated with ImageJ 7.1 open source software (NIH, Bethesda, MD, USA and LOCI, University of Winsconsin-Madison, Madison, WI, USA; https://imagej.nih.gov/ij/). 

### 4.6. Statistical Analysis

The obtained data were pooled and are presented as the mean ± SEM. The statistical analysis was performed using one-way analysis of variance (ANOVA) with Bonferroni’s Multiple Comparison post hoc test using Statistica 12 software (StatSoft Inc., Tulsa, OK, USA). Differences between C group and LD group, as well as C group an HD group were considered statistically significant at *p* ≤ 0.001 (a), *p* ≤ 0.01 (b) and *p* < 0.05 (c). Differences between LD group and HD group were considered statistically significant at *p* ≤ 0.001 (d), *p* ≤ 0.01 (e) and *p* < 0.05 (f).

## 5. Conclusions

The present study, for the first time, describes the occurrence of NRG1 in the neurons of all types of enteric plexuses in the wall of the porcine large intestine. The population of NRG1-positive enteric neurons is relatively large because this substance is present in at least 20% of all enteric neurons within the particular types of the ENS plexuses. Moreover, the obtained results have shown that the administration of BPA causes an increase in the number of enteric neurons immunoreactive to NRG1. The exact mechanisms of the observed changes remain unknown and may be connected with endocrine disruption, as well as neurotoxic, neurodegenerative and pro-inflammatory activity of BPA. On the grounds of the present study, it is difficult to state unambiguously, whether the observed changes are caused more by adverse effects of BPA or are result of adaptive and protective processes. However, considering the relatively well-known participation of NRG 1 in adaptive reactions and neuronal development, the latter of the two options seems more probable. Of course, the exact explanation of this issue requires further research.

## Figures and Tables

**Table 1:** Distribution of NRG1 in the digestive tract of various species.

Species	Localization	References
Human	Myenteric ganglia, in neuronal and glial cells, circular muscular layer in muscular cells and nerve fibers in the distal colon	[3]
	Mucosal layer and enteric ganglia in the colon	[21]
	Myenteric ganglia located in the sigmoid colon	[22]
	The whole colonic wall	[18,23]
	The wall of stomach: in gastric glands under physiological condition, parietal cells in the gastric body, stromal cells of gastric pylorus and in tumor cells located in the stomach	[24,25]
Rhesus monkey	Esophagus: stratified squamous epithelial (SSE) cells bordering the lamina propria	[26]
	Stomach: Chief and parietal cells of gastric glands	
	Small intestine: cells in the lamina propria and enteroendocrine cells	
Mouse	Mucosal layer of the small intestine	[27]
Zebrafish	in the intestinal epithelium and muscularis externa layer serosa layer, or goblet cells	[28]

**Table 2:** The ratio (mean ± SEM%) of the number of NRG1-positive enteric neurons in relation to the total number of PGP9.5-cells: C group—control animals; Ex I—animals treated with BPA at a dose of 0.05 mg/kg b.w./day; Ex II—animals treated with BPA at a dose of 0.5 mg/kg b.w./day.

Part of ENS	Type of Group	Part of Large Intestine		
		Caecum	Ascending Colon	Descending Colon
Myenteric plexus	C group	22.43 ± 0.16%	26.45 ± 0.19%	24.98 ± 0.39%
	Ex I group	32.64 ± 0.17% ^a^	37.38 ± 0.10% ^a^	28.99 ± 0.31% ^a^
	Ex II group	43.10 ± 0.15% ^a,d^	43.33 ± 0.28% ^a,d^	37.11 ± 0.12% ^a,d^
Outer submucous plexus	C group	19.63 ± 0.08%	27.49 ± 0.19%	21.16 ± 0.15%
	Ex I group	24.77 ± 0.29% ^a^	38.75 ± 0.41% ^a^	27.62 ± 0.18% ^a^
	Ex II group	30.62 ± 0.23% ^a,d^	42.62 ± 0.37% ^a,d^	39.64 ± 0.15% ^a,d^
Inner submucous plexus	C group	19.96 ± 0.24%	22.73 ± 0.17%	19.85 ± 0.28%
	Ex I group	29.44 ± 0.33% ^a^	38.17 ± 0.34% ^a^	25.04 ± 0.30% ^a^
	Ex II group	37.85 ± 0.32% ^a,d^	45.55 ± 0.18% ^a,d^	36.79 ± 0.23% ^a,d^

Differences between C group and Ex I group, as well as C group and Ex II group were considered statistically significant at *p* ≤ 0.001 (^a^). Differences between Ex I group and Ex II group were considered statistically significant at *p* ≤ 0.001 (^d^).

**Table 3:** The average surface area (mean ± SEM µm^2^) of NRG1-positive neurons in particular types of the enteric plexuses: C group—control animals; Ex I—animals treated with BPA at a dose of 0.05 mg/kg b.w./day; Ex II—animals treated with BPA at a dose of 0.5 mg/kg b.w./day.

Part of ENS	Type of Group	Part of Large Intestine		
		Caecum	Ascending Colon	Descending Colon
Myenteric plexus	C group	92.05 ± 0.35 µm^2^	91.23 ± 0.27 µm^2^	90.68 ± 0.25 µm^2^
	Ex I group	96.00 ± 0.35 µm^2^ ^a^	96.98 ± 0.30 µm^2^ ^a^	95.02 ± 0.32 µm^2^ ^a^
	Ex II group	103.38 ± 0.25 µm^2^ ^a,d^	101.98 ± 0.34 µm^2^ ^a,d^	104.77 ± 0.27 µm^2^ ^a,d^
Outer submucous plexus	C group	65.20 ± 0.32 µm^2^	64.27 ± 0.27 µm^2^	64.70 ± 0.25 µm^2^
	Ex I group	68.21 ± 0.22 µm^2^ ^a^	67.17 ± 0.17 µm^2^ ^a^	69.25 ± 0.27 µm^2^ ^a^
	Ex II group	70.40 ± 0.18 µm^2^ ^a,d^	68.54 ± 0.21 µm^2^ ^a,e^	72.25 ± 0.27 µm^2^ ^a,d^
Inner submucous plexus	C group	59.25 ± 0.27 µm^2^	60.48 ± 0.22 µm^2^	58.03 ± 0.15 µm^2^
	Ex I group	61.94 ± 0.34 µm^2^ ^a^	63.44 ± 0.22 µm^2^ ^a^	60.44 ± 0.26 µm^2^ ^a^
	Ex II group	66.23 ± 0.30 µm^2^ ^a,d^	67.13 ± 0.29 µm^2^ ^a,d^	65.33 ± 0.27 µm^2^ ^a,d^

Differences between C group and Ex I group, as well as C group and Ex II group were considered statistically significant at *p* ≤ 0.001 (^a^). Differences between Ex I group and Ex II group were considered statistically significant at *p* ≤ 0.001 (^d^) and *p* ≤ 0.01 (^e^).

**Table 4:** The average total number (mean ± SEM) of PGP 9.5-positive cells in particular types of the enteric plexuses: C group—control animals; Ex I—animals treated with BPA at a dose of 0.05 mg/kg b.w./day; Ex II—animals treated with BPA at a dose of 0.5 mg/kg b.w./day.

Part of ENS	Type of Group	Part of Large Intestine		
		Caecum	Ascending Colon	Descending Colon
Myenteric plexus	C group	1086.00 ± 5.89	1077.20 ± 5.99	1088.80 ± 3.54
	Ex I group	1054.20 ± 5.95 ^b^	1051.20 ± 4.63 ^b^	1063.20 ± 5.61 ^b^
	Ex II group	1006.60 ± 5.36 ^a,d^	996.20 ± 3.40 ^a,d^	1025.20 ± 5.95 ^a,d^
Outer submucous plexus	C group	888.20 ± 6.59	870.00 ± 4.24	879.20 ± 6.08
	Ex I group	865.80 ± 2.48 ^c^	868.60 ± 5.86	855.20 ± 4.07 ^c^
	Ex II group	850.00 ± 6.75 ^d^	846.80 ± 6.89 ^c^	829.20 ± 5.08 ^a,e^
Inner submucous plexus	C group	761.60 ± 5.35	767.40 ± 4.68	787.40 ± 5.84
	Ex I group	717.80 ± 5.87 ^a^	701.00 ± 4.32 ^a^	731.60 ± 4.50 ^a^
	Ex II group	690.00 ± 4.24 ^a,e^	680.00 ± 4.11 ^a,f^	706.00 ± 3.48 ^a,e^

Differences between C group and Ex I group, as well as C group and Ex II group were considered statistically significant at *p* ≤ 0.001 (^a^), *p* ≤ 0.01 (^b^) and *p* < 0.05 (^c^). Differences between Ex I group and Ex II group were considered statistically significant at *p* ≤ 0.001 (^d^), *p* ≤ 0.01 (^e^) and *p* < 0.05 (^f^).
